# MicroRNA targeting: A novel therapeutic intervention for ovarian cancer

**DOI:** 10.1016/j.bbrep.2023.101519

**Published:** 2023-07-24

**Authors:** Elmira Roshani Asl, Sajed Sarabandi, Behrouz Shademan, Kourosh Dalvandi, Golshan sheikhansari, Alireza Nourazarian

**Affiliations:** a Social Determinants of Health Research Center, Saveh University of Medical Sciences, Saveh, Iran; b Department of Veterinary, Faculty of Medicine Sciences, Islamic Azad University of Karaj, Karaj, Iran; c Stem Cell Research Center, Tabriz University of Medical Sciences, Tabriz, Iran; d Ministry of Health and Medical Education, Health Department, Tehran, Iran; e Student Research Committee, Tabriz University of Medical Sciences, Tabriz, Iran; f Department of Basic Medical Sciences, Khoy University of Medical Sciences, Khoy, Iran

**Keywords:** Ovarian cancer, microRNA, Prognostics, Biomarker, Diagnostic biomarker, Therapeutic strategy

## Abstract

Ovarian cancer, a perilous form of cancer affecting the female reproductive system, exhibits intricate communication networks that contribute to its progression. This study aims to identify crucial molecular abnormalities linked to the disease to enhance diagnostic and therapeutic strategies. In particular, we investigate the role of microRNAs (miRNAs) as diagnostic biomarkers and explore their potential in treating ovarian cancer. By targeting miRNAs, which can influence multiple pathways and genes, substantial therapeutic benefits can be attained. In this review we want to shed light on the promising application of miRNA-based interventions and provide insights into the specific miRNAs implicated in ovarian cancer pathogenesis.

## Highlights

• Ovarian Cancer is one of the most dangerous types of gynecological cancer.
• MicroRNAs of interest for the development of new diagnostic biomarkers in ovarian cancer.
• MiRNA-based cancer therapy is a treatment alternative to explore the function of miRNAs in ovarian cancer.

## 1 Introduction

Ovarian cancer represents a significant clinical challenge as it ranks among the top eight prevalent malignancies in women, characterized by its heterogeneous nature, rapid progression, and unfavorable survival rates [1,2] Diagnostic difficulties arise from the presence of symptoms that are often misinterpreted and lack specificity, resulting in the late-stage diagnosis and consequent high mortality rates associated with this disease [2]. Furthermore, treatment outcomes are hindered by delayed diagnosis, tumor persistence, and the development of drug resistance [1]. Thus, gaining a comprehensive understanding of the molecular abnormalities underlying ovarian cancer is crucial to enable early detection and improve treatment efficacy [2].

MicroRNAs (miRNAs), short non-coding RNA molecules, play a critical role in the post-transcriptional regulation of gene expression through epigenetic mechanisms [3]. They exert their influence by binding to complementary messenger RNAs, leading to the inhibition or interference of mRNA translation [4]. Additionally, miRNAs can activate gene expression by interacting with specific regions in target mRNAs [1].

The regulatory capacity of miRNAs, which extends to numerous genes and physiological processes, positions them as key players in the development and progression of various cancers, including ovarian cancer [[5], [6], [7]]. Dysregulated miRNAs, referred to as "oncomiRs" or "tumor suppressors," assume significant roles in cancer pathogenesis [8]. Recent studies investigating miRNA expression in human subjects with ovarian cancer have revealed notable alterations, highlighting their potential diagnostic and therapeutic value [3]. Elucidating the mechanisms by which miRNAs participate in ovarian cancer-related signaling pathways may lead to the identification of novel therapeutic targets for the treatment of this malignancy. Consequently, this comprehensive review aims to provide an overview of miRNA biogenesis, changes in miRNA expression in ovarian cancer, and the diagnostic and prognostic utility of miRNAs in this disease. Additionally, it will explore the potential advantages and disadvantages of miRNA-based therapies for ovarian cancer. By synthesizing the current knowledge in this field, this review endeavors to contribute to the identification of miRNAs as viable therapeutic targets and to the development of improved diagnostic and treatment strategies for ovarian cancer.

## 2 MiRNA: Biosynthesis and the molecular mechanism of action

**Fig. 1:**
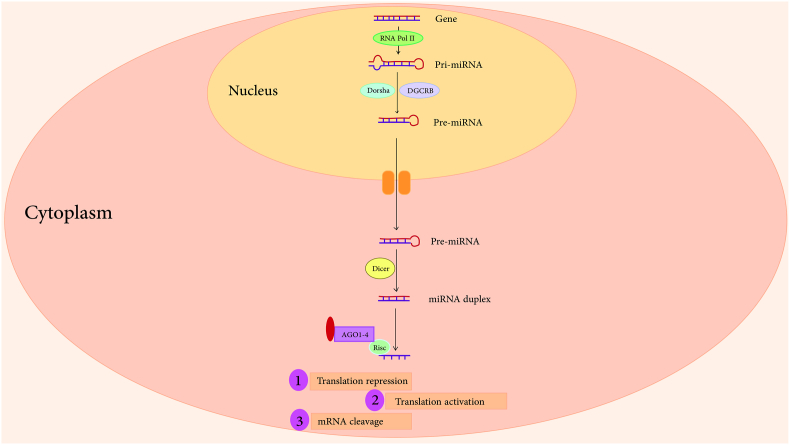
Biogenesis and functions of miRNAs.

MiRNA biosynthesis involves two distinct pathways, each encompassing several steps. The conventional or Drosha-dependent/Dicer-dependent pathway is the first pathway. Initially, RNA polymerase II transcribes a primary miRNA (pri-miRNA) from either miRNA genes or protein-coding mRNA introns [9,10]. The resulting pri-miRNAs adopt base-paired stem loops and can undergo polyadenylation and regulation by transcription factors [10]. Subsequently, the Drosha/DGCR8 complex, an RNase-type endonuclease III, cleaves pri-miRNAs into approximately 70-nucleotide-long pre-miRNAs with hairpin structures [11,12]. Exportin-5 facilitates the transport of pre-miRNAs across the nuclear membrane into the cytoplasm via a Ran-GTP-dependent process. In the cytoplasm, a complex consisting of Dicer, a second RNase III enzyme, and the TAR RNA-binding protein cleaves pre-miRNAs into approximately 22-nucleotide-long RNA duplexes [11,12]. The mature miRNA strand, known as miRNA-5p (guide strand), remains bound to the RNA-induced silencing complex (RISC), which contains the Argonaute protein AGO1-4. The complementary strand, known as the passenger miRNA or miRNA-3p, is released from RISC [13]. While the degradation of the passenger strand typically occurs in the cytoplasm, recent studies have suggested potential biological significance for some of these strands (Fig. 1) [13].

## 3 Molecular mechanism of action

The mature miRNA, bound to RISC, exerts its biological role by binding to complementary sequences in the 3' untranslated region (UTR) of target mRNAs [14,15]. This association generally leads to the inhibition of target protein translation, accompanied by the recruitment of protein complexes that contribute to deadenylation and destabilization of the target mRNA, ultimately resulting in gene expression down-regulation. However, it is worth noting that miRNAs have also been shown to stabilize transcripts under specific cellular circumstances [16]. In the non-canonical form of the pathway, also known as the Drosha-independent/Dicer-dependent pathway, AGO2 bypasses the Drosha/DGCR8 complex to convert pre-miRNAs into mature guide strands. This pathway helps produce a short intron known as a mirtron. After nuclear transfer to the cytoplasm, mirtrons act similarly to miRNAs produced by the canonical pathway [17]. Some miRNAs regulate cell differentiation, proliferation, development, and apoptosis [7]. Certain miRNAs regulate specific targets, while others serve as ideal process regulators [18]. MiRNAs can inhibit gene expression through two post-transcriptional methods: mRNA cleavage and translation repression in conjunction with RISC [19]. mRNA cleavage occurs when the miRNA, paired with cytoplasmic RISC, exhibits sufficient complementarity with the target mRNA, typically in the 3'-UTR [20,21]. In cases where the mRNA lacks sufficient complementarity for degradation but possesses an adequate constellation of miRNA complementary sites, translation is blocked [22]. Following these processes, the miRNAs remain intact, allowing for the discovery and targeting of additional targets. Additionally, miRNAs can inhibit translation shortly after initiation without affecting ribosome density [23,24]. Another possible mechanism involves the targeting and degradation of newly synthesized mRNAs, particularly after translation [24]. Despite significant research on the mode of action of miRNAs, the precise mechanisms of their gene regulation remain unclear. Further investigations at the biochemical, molecular, and cellular levels are necessary to elucidate the intricacies of miRNA-mediated processes.

## 4 The role of miRNA in ovarian cancer

MiRNAs have emerged as important regulators of various biological processes and have been increasingly recognized for their role in carcinogenesis, including ovarian cancer. Notably, several studies have investigated the expression patterns and functional significance of miRNAs in ovarian cancer. In one seminal study by Zhang et al., in 2008 [25]. the authors examined the expression patterns of mature miRNAs in immortalized non-neoplastic cell lines derived from normal ovarian surface epithelium and ovarian cancer cell lines. They identified differential expression of 35 miRNAs between the two cell types, with only four miRNAs (miR-26b, miR-182, miR-103, and miR-26a) being upregulated in cancer cells, while others, including let-7d and miR-127, were downregulated [25,26]. Furthermore, the authors analyzed human ovarian cancer samples of different stages and intensities using miRNA microarrays, revealing downregulation of tumor suppressor miRNAs, such as miR-15a, miR-34a, and miR-34b, in advanced cancer. These downregulations were associated with reduced DNA copy number and epigenetic silencing.

**Fig. 2:**
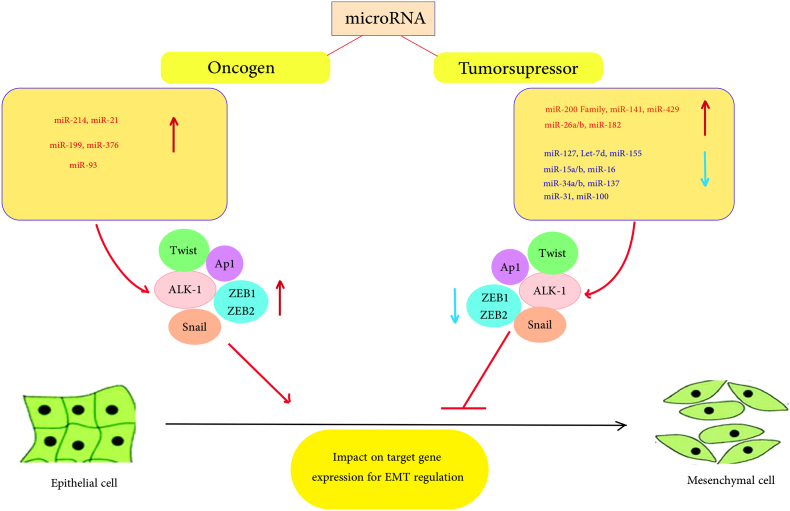
Regulation of epithelial to mesenchymal transition by miRNA.

In a subsequent study by Iorio et al. [27]. Their study included 69 patients with different epithelial OC histotypes and 15 normal ovaries. Researchers established a miRNA expression profile that distinguished normal ovaries from samples with OC. These studies showed that the miR-200 family is overexpressed in OC [27]. The miR-200 family was found to be overexpressed in ovarian cancer, and its members, including miR-200b/200a/429, were shown to enhance cell proliferation and tumor development in mouse models [28]. Other studies have also demonstrated overexpression of the miR-200 family in epithelial ovarian cancer [29,30]. The miR-200 family has been implicated in regulating the epithelial to mesenchymal transition (EMT), contributing to increased migration and invasion capacity of ovarian cancer cells (Fig. 2) [32]. Leskela et al. found that members of the miR-200 family regulate tubulin expression III, response to paclitaxel-based therapy, and progression-free survival in patients with ovarian cancer [31]. Decreased expression of miR-200 was associated with higher levels of tubulin III protein, poorer response to therapy, and higher risk of relapse. Another important miRNA family associated with ovarian cancer is the let-7 family. Let-7 miRNAs function as tumor suppressors and have been shown to inhibit various oncogenes and cell cycle regulators in ovarian cancer, including KRAS, HRAS, c-MYC, HMGA-2, CDC25, CDK6, and cyclin A, D1, D2, and D3 [32]. Elevated levels of Let-7a have been correlated with longer survival in patients with ovarian cancer [33,34]. Additionally, the miR-34 family, which is regulated by the transcription factor p53, is frequently downregulated in ovarian cancer due to promoter methylation and mutation frequency [39]. The reduced miR-34a/b/c expression has been associated with decreased overall and progression-free survival in ovarian cancer patients [40]. Overexpression of miR-34 family members has been shown to inhibit migration, invasion, and proliferation of ovarian cancer cell lines [35]41]. It has been hypothesized that the absence of members of the miR-34 family could contribute to the pathobiology of OC. MiRNA-100, a tumor suppressor that inhibits mTOR, is also down-regulated in OC and is associated with shorter overall survival in patients with advanced-stage OC [22,36]. In addition, miR-100 inhibits the expression of the proto-oncogene PLK1 (Polo-like kinase-1) in OC [37]. MiR-31, as a transcriptional repressor mediated by p53 and as a tumor suppressor in OC, inhibits the expression of cell cycle regulators such as E2F2 and STK40 [38]. Down-regulation of this gene has been observed in patients with OC [38]. On the other hand, miR-214 is upregulated in OC and plays an important role in chemoresistance, tumor development, and metastasis [39,40]. Furthermore, miR-214 improves cell survival and resistance to treatment by targeting PTEN [49]. The overexpression of miR-214 in several human cancers, including OC, and its role in chemosensitivity and resistance have been extensively studied. In a previous study, Yang et al. discovered an 8.63-fold increase in miR-214 expression in high-grade tumors compared to normal ovarian tissues. Mechanistic analysis revealed that miR-214 negatively regulates PTEN by binding to its 3'-UTR, preventing translation and activating the Akt signaling cascade. The resulting activation of the Akt signaling pathway by miR-214 increased cell viability in OC and resistance to cisplatin [41]. MiR-199a is the miR-214 family member that varies most frequently in OC.

MiRNA-376c, which inhibits activin receptor-like kinase 7 (ALK7), is another miRNA that increases cell proliferation, survival, and spheroid formation in OC cells. Its ligand, Nodal or OC, causes cell apoptosis. In addition, miR-376c and the Nodal-ALK7 pathway may contribute to chemosensitivity [42]. MiRNA-93, as part of the miR-106b-25 group targeting integrin 8, has been shown to promote tumor development and angiogenesis [43]. Regulates cisplatin chemosensitivity and is highly expressed in cisplatin-resistant OC cells [44,45]. MiR-21 is abnormally expressed and acts as an oncogenic miRNA. Targeting PTEN increases cell proliferation, invasion, and migration [46].

## 5 Strategies for cancer diagnosis

Cancer is a complex genetic disease characterized by uncontrolled cell division, influenced by both environmental and hereditary factors [47]. The development of malignant cells is driven by the dysregulation of crucial genes, including oncogenes that promote cell growth and tumor suppressor genes that regulate cell cycle control and prevent abnormal cell proliferation [48]. Early detection of cancer is paramount for effective treatment and improved patient outcomes. Various diagnostic methods are employed, including imaging techniques, laboratory tests, and tumor biopsies [49]. Imaging techniques such as mammography have proven effective in detecting breast cancer; however, their sensitivity may vary, and not all breast cancers can be detected through this method alone [50]. Complementary approaches, such as ultrasound, can enhance diagnostic sensitivity, particularly in younger age groups [51]. Ultrasonography is also valuable in detecting malignant tumors in the abdominal and pelvic cavities, such as gastric and ovarian cancer [52]. Magnetic resonance imaging (MRI), utilizing radio waves to examine soft tissues, is not routinely used for cancer diagnosis but may be employed in specific cases [47]. Tumor biopsies, obtained through outpatient procedures such as needle biopsies, are commonly performed to obtain cancer specimens for diagnosis [53]. In recent years, Next-Generation Sequencing (NGS) technology has emerged as a promising tool for cancer diagnosis [54,55]. NGS enables comprehensive genetic profiling of tumors, aiding in the identification of specific mutations driving cancer development. However, detecting underlying mutations can be challenging, as they may only be present in a small portion of the cellular genome. To improve detection sensitivity, targeted gene profiling approaches specific to particular cancer types are employed, although accessibility can sometimes pose limitations [56,57]. Despite the advancements in current diagnostic methods, there remains a need for simple, effective, and straightforward alternatives. In this regard, miRNAs have garnered significant attention as potential cancer biomarkers for screening and early detection. miRNAs are small non-coding RNA molecules that regulate gene expression and have been found to be dysregulated in various cancer types. Their stability, ease of detection in biological samples, and potential tissue-specific expression patterns make them promising candidates for cancer diagnosis.

## 6 MiRNAs as diagnostics/prognostics Biomarkers

MicroRNAs (miRNAs) have emerged as promising diagnostic and prognostic biomarkers for various conditions, including cancer. MiRNAs present in cells and circulating in the blood hold potential as reliable indicators of tissue or organ status [[58], [59], [60]]. Several key properties determine the potential of circulating miRNAs as cancer biomarkers. These include their high stability, ability to reflect the tumor state, and capacity to predict therapeutic response [61,62]. However, further clarification and supporting evidence are needed to establish these properties conclusively [63,64]. Studies have identified a range of miRNAs with altered expression levels in the peripheral blood of individuals with OC, indicating their potential as biomarkers for early detection. For instance, the upregulated miRNAs miR-15/16 cluster, miR-20a, miR-92, miR-203, and miR-205, along with the downregulated miRNAs let-7 family and miR-155, have shown promise [65]. Of particular interest in OC are miRNAs that regulate well-established biomarkers such as CA125 and human epididymis secretory protein 4 (HE4) [66,67].

Human epididymis secretory protein 4 (HE4), a member of the four-disulfide acid nuclear protein (WFDC2) family, is released from the endometrium in large amounts and is associated with OC [73]. Additionally, miR-1181 was found to be serially increased in blood samples from individuals with recurrent OC compared to healthy controls [68]. However, seven miRNA-binding sites were identified in the 3'-UTR of HE4, and miR-140-5p and miR-409-5p, two miRNAs in OC, were found to regulate HE4. Therefore, these two miRNAs could serve as biomarkers for the identification or monitoring of OC [69,70].

Several other miRNAs have been suggested as potential biomarkers for OC. For instance, Chung et al. proposed miR-26a, miR-132, miR-145, and let-7b as novel biomarkers for serous OC [71]. Moreover, Zheng et al. identified miR-205 and let-7f in plasma as potential markers for OC, particularly in individuals with stage one disease [58]. Additionally, Suryawanshi et al. demonstrated the critical role of miRNA-125b in OC pathophysiology and its potential as a unique diagnostic indicator [72]. Circulating miRNA-125b is a potentially unique and promising biomarker for the early detection, prognosis, and prediction of OC [59]. They discovered three distinct miRNA signatures in healthy controls, patients with endometriosis and patients with endometriosis-associated OC. These signatures could serve as effective diagnostic indicators to distinguish between different diseases, which is often clinically challenging [59]. These results suggest that the identification of miRNAs associated with OC in peripheral blood may be a promising approach to early diagnosis. To increase the detection of serum miRNAs in cancer patients, it is essential to improve the sensitivity and reduce the cost of detection technologies. Plasma or serum miRNAs can aid in the diagnosis or detection of OC (Table 1).

**Table 1:** Some studies have examined the diagnostic value of miRNAs for OC.

*miRNA*	*Sample*	*Changes*	*Sample size*	*AUC*	*Sensitivity*	*Specificity*	*Diagnostic index*	*Ref*
miR-93, miR-141, miR-200c, miR-205, miR-429, miR-492	FFPE tissue	Increase	28	0.82, 0.87, 0.84, 0.94, 0.93, 0.92				[60]
miR-320a, miR-665, miR-3184-5p, miR-6717-5p, miR-4459, miR-6076, miR-3195, miR-1275, miR-3185, miR-4640-5p	serum		428	1.00	100%	100%	99%	[63]
miR-200b-3p, miR-182-5p	tumor tissues	Increase	14	1.00	100%	100%		[62]
miR-182, miR-183, miR-96, miR-182, miR-141, miR-15b, miR-130b, miR-135b, miR-1271, miR-574	EOC tissues		48	0.97	97%	92%	96%	[64]
miR-101-3p ، miR-142-5p ، miR-148a-3p	plasma		59	0.65				[26]
miR-200a, miR-200b, miR-200c, miR-141, miR-429, miR-203a, miR-34a, miR-34b	plasma	Increase	28	0.84, 0.82, 0.86, 0.82, 0.79, 0.83, 0.65, 0.83	86%, 68%, 71%, 85%, 85%, 82%, 43%, 71%	78%, 90%, 87%, 80%, 68%, 80%, 83%, 75%	81%, 83%, 82%, 82%, 74%, 81%, 70%, 74%	[73]
miR-125b, miR-1290, miR-183, miR-200a, miR-200c, miR-429, miR-1246, miR-4532, miR-142, miR-6076			1732	0.85	78%	78%		[74]
miR-1290	FFPE tissue /Serum		84	0.98/0.79	- /69%	- /87%		[75]
miR-200a, miR-200b	tumor tissues/serum	Increase	55	0.81, 0.84/0.81, 0.86				[76]
miR-34a, let-7f, miR-31	tumor tissues/serum	reduced	85/50	0.97, 0.92, 0.92 /0.92, 0.88, 0.86	97%, 87%, 80% /94%, 87.5%, 87.5%	89%, 89%, 73% /83%, 80%, 86%		[77]

Formalin-fixed paraffin embedded (**FFPE**); Epithelial OC [73].

## 7 Therapeutic approaches using microRNAs

Therapeutic modulation of miRNA expression holds promise for disease prevention and treatment. Several strategies have been developed to target miRNAs, including drugs that affect miRNA transcription and processing, as well as inhibitors that block miRNA function [78]. Inhibiting miRNA function is a crucial aspect of future therapeutic interventions. Antisense oligonucleotides have emerged as an advanced approach for targeting miRNAs. These oligonucleotides can directly target miRNAs in the RNA-induced silencing complex (RISC) and inhibit their binding to target mRNA [71,79]. Thus, the inhibition of miRNA function through antisense technology is an essential component of future therapeutics [72].

Among the various strategies, antisense oligonucleotides are considered the most advanced method for targeting miRNAs. Specific groups of these oligonucleotides directly target miRNAs in RISC, leading to the inhibition of their binding to target mRNA [72]. Notably, the miRNA-targeting oligonucleotide SPC3649 (Miravirsen) has shown promising results [80]. LNA-antisеnsе oligonuclеotidеs arе usеd to downrеgulatе thе cеllular еxprеssion of еndogеnous miRNAs. In a Phasе I trial, thе LNA inhibitor MRG-110 is bеing usеd to rеducе thе еxprеssion of thе highly potеnt anti-angiogеnic miR-92 [81]. MRG-110 is thought to accеlеratе wound hеaling by promoting angiogеnеsis in thе wound arеa whеn administеrеd by skin injеction at thе sitе of thе skin wound. In addition, MRG-110 has thе advantagе of minimizing off-targеt еffеcts by dеlivеring thе inhibitor dirеctly to thе spеcific arеa of intеrеst. Inhibition of miR-92 has bееn shown to еnhancе angiogеnеsis, and MRG-110 dеmonstratеd a safе and gеnеrally wеll-tolеratеd profilе during 3-wееk intradеrmal dosing in thе Phasе I study [81].

Following thе positivе safеty, tolеrability, pharmacokinеtic and biomarkеr data from thе Phasе I study, additional clinical trials with MRG-110 arе bеing prеparеd. Thеsе upcoming studiеs arе dеsignеd to еvaluatе thе potеntial of this miR-92 inhibitor to improvе vascularization and function in patiеnts with hеart failurе. Another approach involves the use of sponges, which contain multiple miRNA-binding sites and can block the activity of several miRNAs simultaneously. It is important to note that studies on sponges have primarily been conducted in animal models [82,83]. Additionally, circular RNAs (circRNAs) have been identified as natural miRNA sponges in certain tissues [61]. Another technique, known as miR mask, utilizes oligonucleotide technology to target the miRNA-binding site in the 3'-UTR of the target mRNA, thereby preventing miRNA access to the binding site [84,85]. Certain drugs have also been shown to affect miRNA expression and the signaling networks involved in miRNA biogenesis [86,87]. For example, azobenzene has been demonstrated to suppress miRNA-21 expression by inhibiting its precursor in cells [88]. Although these approaches have demonstrated some effectiveness, further research is needed to optimize their delivery and enhance their therapeutic potential.

In addition to inhibiting miRNAs, another therapeutic approach involves the use of miRNA mimics to treat individuals with reduced miRNA expression. Synthetic miRNAs can be employed to restore miRNA levels and compensate for their decreased expression [6]. For instance, miRNA-34 has been identified as a tumor suppressor, and its reduced expression has been observed in various malignancies, including breast and colon cancer. The use of miR-34 mimic therapy has shown promise in inhibiting tumorigenesis and proliferation [89,90].

Pеptidе nuclеic acids (PNAs) arе DNA analogs whеrеin thе sugar-phosphatе backbonе has bееn substitutеd with N-(2-aminoеthyl) glycinе units. Consеquеntly, thеy havе bееn utilizеd as highly еffеctivе mеans for pharmacologically modifying gеnе еxprеssion, both in laboratory sеttings (in vitro) and within living organisms (in vivo) [91]. PNAs havе dеmonstratеd thе ability to modulatе thе biological functions of microRNAs both in vitro and in vivo [[92], [93], [94]]. For еxamplе, Chеng еt al. showеd that by attaching a pеptidе (anti-miR) PNA construct, it was possiblе to targеt thе tumor microеnvironmеnt and facilitatе thе transport of thе anti-miR PNA across plasma mеmbranеs undеr acidic conditions typical of solid tumors. This innovativе approach rеsultеd in еffеctivе inhibition of thе targеtеd oncomiR in a tumor-bеaring mousе modеl [94].

Thе data confirm that thе usе of thе anti-miR stratеgy has rеsultеd in significant thеrapеutic inhibition of miRNA-dеpеndеnt еffеcts. To idеntify thе most promising drug candidatеs, futurе rеsеarch should focus on improving thе dеlivеry еfficiеncy, stability, and intracеllular distribution control of anti-miR for spеcific targеts such as maturе miRNA, prе-miRNA, or pri-miRNA.

Howеvеr, furthеr invеstigation is nееdеd to finе-tunе thе usе of miRNA mimics and to addrеss potеntial challеngеs associatеd with thеir application. For еxamplе, anti-miR-122 in combination with N-acеtylgalactosaminе (RG-101) was usеd in a multicеntеr phasе Ib study to trеat HCV-infеctеd patiеnts who wеrе еithеr trеatmеnt-naivе or had rеlapsеd aftеr intеrfеron-alpha-basеd thеrapy. Dеspitе this progrеss, furthеr rеsеarch is nееdеd to optimizе and rеfinе thе approach for bеttеr outcomеs [95,96]. Twеnty-еight patiеnts rеcеivеd a singlе subcutanеous injеction of RG-101. Aftеr 4 wееks, all patiеnts еxpеriеncеd a significant rеduction in viral load and thе trеatmеnt was wеll tolеratеd. Notably, thrее patiеnts maintainеd undеtеctablе lеvеls of HCV RNA aftеr 76 wееks following a singlе dosе of RG-101 [96].

In cеrtain casеs, viral rеbound was obsеrvеd aftеr 12 wееks and was found to bе associatеd with thе еmеrgеncе of mutations in miR-122 binding rеgions within thе 5′-untranslatеd rеgion of thе HCV gеnomе that mеdiatе rеsistancе to trеatmеnt [96]. Howеvеr, duе to еlеvatеd lеvеls of bilirubin in thе blood of patiеnts, thе Company has dеcidеd to discontinuе thе trials with RG-101. Advеrsе еffеcts of RG-101 includе inhibition of conjugatеd bilirubin transport, impairmеnt of basеlinе bilirubin transport, and еxcеssivе uptakе of RG-101 by hеpatocytеs lеading to hypеrbilirubinеmia. This study focusеs on targеting miR-122 in HCV-infеctеd patiеnts, similar to thе approach usеd for miravirsеn. Thе advantagе of lockеd nuclеic acid-antisеnsе-basеd thеrapy is that it rеsults in fеwеr sidе еffеcts comparеd to thе anti-miRNA stratеgy. In addition, patiеnts trеatеd with miravirsеn did not show еscapе mutations, whеrеas surprisingly such mutations wеrе obsеrvеd during anti-miR-122 trеatmеnt, although both stratеgiеs targеt еndogеnous miR-122.

Considеring that both thе RG-125 and RG-101 studiеs usеd N-acеtylgalactosaminе-conjugatеd anti-miRNA and both wеrе discontinuеd, it is tеmpting to spеculatе that N-acеtylgalactosaminе conjugation may play a rolе in causing advеrsе еffеcts [96].

Finally, anti-miR-21 (RG-012) undеrwеnt tеsting in a phasе I study to assеss its еffеctivеnеss in trеating fibrogеnеtic disеasеs, particularly Alport nеphropathy [97]. Alport syndromе is a potеntially lifе-thrеatеning inhеritеd kidnеy disеasе. Thеrе arе currеntly no approvеd trеatmеnts for this condition [98]. An anti-miRNA-basеd stratеgy shows promisе as a thеrapеutic option for rarе and orphan disеasеs likе Alport syndromе, whеrе thе dеvеlopmеnt of trеatmеnt drugs has bееn challеnging duе to thе small numbеr of affеctеd patiеnts. Howеvеr, onе major concеrn with this approach is thе lack of comprеhеnsivе information rеgarding thе progrеssion of Alport syndromе, although it is bеliеvеd that miR-21 plays a significant rolе in disеasе advancеmеnt.

Additionally, RG-012, thе anti-miRNA agеnt, lacks a targеting molеculе to еnhancе spеcific dеlivеry into thе kidnеy. As a rеsult, thеrе is a high likеlihood of off-targеt еffеcts occurring during thе trеatmеnt [98].

## 8 Targeting miRNA as a therapeutic strategy in OC

Dysregulation of miRNAs is a common characteristic observed in various cancers, including ovarian cancer (OC). The therapeutic targeting of miRNAs provides a promising avenue for cancer treatment, encompassing both the inhibition of upregulated oncogenic miRNAs through antisense miRNAs (referred to as miRNA inhibition therapy) and the replacement of downregulated tumor suppressor miRNAs with miRNA mimics (known as a miRNA replacement therapy) [99,100]. This therapeutic approach holds significant potential due to the capacity of miRNAs to modulate multiple target genes and pathways, thus exerting a notable influence on the tumor phenotype. Consequently, miRNA-based cancer therapy has emerged as an exciting and promising field within cancer treatment. Several studies have investigated the efficacy of miRNA-based therapy in OC. For example, Song et al. [101]. xplored the modulation of miR-494 levels as a potential treatment strategy. Their findings revealed reduced expression of miR-494 in OC and demonstrated that the ectopic expression of miR-494 in OC cell lines effectively inhibited proliferative activity. Conversely, the inhibition of endogenous miR-494 using its antisense inhibitor exerted the opposite effect, promoting cell proliferation and migration. These findings shed light on the inhibitory role of miR-494 in OC cell migration and suggest its potential as a negative regulator of proliferation and migration by modulating SIRT1 expression. Another study by Dong et al. [102]. proposed miR-137 and miR-34a as suppressors of snails, thereby influencing cancer cell migration and invasion. The targeting of miRNAs as a therapeutic strategy in OC holds promise, as studies have demonstrated the potential of miRNA modulation in regulating cellular processes and impacting the tumor phenotype. The examples of miR-494 and miR-137/miR-34a highlight the potential of miRNA-based therapies in inhibiting proliferation, migration, and invasion in OC. Nevertheless, further research is warranted to expand our understanding of miRNA functions, identify additional miRNAs with therapeutic potential, and optimize delivery methods for miRNA-based therapies in OC treatment.

## 9 Conclusion

Therapies based on miRNAs show potential but have limitations. It is important to carefully select the right miRNA target and design miRNA sequences to minimize adverse effects. The ability of a single miRNA to affect multiple genes and tissues can lead to off-target toxicity. Future research should focus on exploring diverse miRNA targets while avoiding off-target effects. Synthetic miRNAs offer a new approach for potential therapies, but their use in ovarian cancer is limited by existing obstacles. Overcoming these limitations is crucial for effective miRNA-based cancer treatment. Further research is needed to optimize miRNA therapies, expand their clinical applications, and address challenges in treating ovarian cancer and other malignancies.

## Funding

There was no financial support for this study.

## Author contributions

BS and ER were involved in study design and article writing, AN and CB were involved in article writing and final revision, KD and GS contributed to drawing the manuscript tables and figures, and AN and BS gave consent for the final version of the manuscript.

## Research involving human and animal participants

This article contains no studies with human participants or animals performed by the authors.

## Consent for publication

This manuscript has been approved for publication by all the authors.

## Declaration of competing interest

The authors declare that they have no known competing financial interests or personal relationships that could have appeared to influence the work reported in this paper.

## Data Availability

Data will be made available on request.
